# Pterostilbene accelerates wound healing by modulating diabetes-induced estrogen receptor β suppression in hematopoietic stem cells

**DOI:** 10.1093/burnst/tkaa045

**Published:** 2021-02-22

**Authors:** Weiguo Xie, Xueqing Zhou, Weigang Hu, Zhigang Chu, Qiongfang Ruan, Haimou Zhang, Min Li, Hongyu Zhang, Xiaodong Huang, Paul Yao

**Affiliations:** 1 Institute of Burns, Tongren Hospital of Wuhan University (Wuhan Third Hospital), Wuhan 430060, China; 2 State Key Lab of Biocatalysis and Enzyme Engineering, School of Life Sciences, Hubei University, Wuhan, 430062, China; 3 Department of Hematology, Peking University Shenzhen Hospital, Shenzhen, 518036, China

**Keywords:** Hematopoietic stem cells, Inflammation, Oxidative stress, Pterostilbene, Wound healing

## Abstract

**Background:**

Delayed wound healing is one of the major complications of diabetes mellitus and is characterized by prolonged inflammation, delayed re-epithelialization and consistent oxidative stress, although the detailed mechanism remains unknown. In this study, we aimed to investigate the potential role and effect of pterostilbene (PTE) and hematopoietic stem cells (HSCs) on diabetic wound healing.

**Methods:**

Diabetic rats were used to measure the epigenetic changes in both HSCs and peripheral blood mononuclear cells (PBMCs). A cutaneous burn injury was induced in the rats and PTE-treated diabetic HSCs were transplanted for evaluation of wound healing. In addition, several biomedical parameters, including gene expression, oxidative stress, mitochondrial function and inflammation in macrophages, were also measured.

**Results:**

Our data showed that PTE had a much stronger effect than resveratrol on accelerating diabetic wound healing, likely because PTE can ameliorate diabetes-induced epigenetic changes to estrogen receptor β promoter in HSCs, while resveratrol cannot. Further investigation showed that bone marrow transplantation of PTE-treated diabetic HSCs restores diabetes-induced suppression of estrogen receptor β and its target genes, including nuclear respiratory factor-1 and superoxide dismutase 2, and protects against diabetes-induced oxidative stress, mitochondrial dysfunction and elevated pro-inflammatory cytokines in both PBMCs and macrophages, subsequently accelerating cutaneous wound healing.

**Conclusions:**

HSC may play an important role in wound healing through transferring epigenetic modifications to subsequent PBMCs and macrophages by differentiation, while PTE accelerates diabetic wound healing by modulating diabetes-induced epigenetic changes in HSCs. Thus, PTE may be a novel therapeutic strategy for diabetic wound healing.

Highlights

- Hematopoietic Stem Cells (HSC) play an important role in wound healing.
- HSC transfers epigenetic modifications to PBMC and macrophages by differentiation.
- Pterostilbene accelerates diabetic wound healing by modulating epigenetic changes.

## Background

Cutaneous wound healing involves a complicated process, with steps including inflammation, angiogenesis/proliferation and maturation/remodeling [1, 2]. Diabetes delays wound healing due to its interruption of the proper healing process through a complicated defective regulation of molecular and cellular events [3]. Delayed wound healing is one of the major complications of diabetes mellitus, potentially leading to issues including diabetic foot ulcers and limb amputations [4, 5], and is characterized by prolonged inflammation, delayed re-epithelialization and consistent oxidative stress [6–9]. Despite the heavy social burden and severe clinical consequences, the underlying mechanisms for diabetic wound healing remain largely unknown [10].

Stem cells are considered to be a powerful tool for wound healing due to their many therapeutic effects, including low immunogenicity and capacity for self-renewal and differentiation [1, 2]. It has been recently reported that hematopoietic stem cells (HSCs) are involved in diabetic wound healing through oxidative stress-mediated epigenetic changes [9, 11, 12], which subsequently impairs differentiation of HSCs into macrophages [13]. HSCs are mostly located in the bone marrow and are responsible for generation of blood cells, monocytes/macrophages and immune cells [14, 15]. Thus, we hypothesize that macrophages [13] and peripheral blood mononuclear cells (PBMCs) may inherit the same epigenetic modifications [16] from HSCs as those that are triggered by diabetes or hyperglycemia exposure, which subsequently impairs wound healing through abnormal gene expression, immune dysfunction and elevated cytokine levels [13, 17].

It has been reported that estrogen receptor regulates mitochondrial function through nuclear respiratory factor-1 (NRF1). NRF1 can interact with coactivator PGC1α to regulate nuclear-encoded mitochondrial genes [18]; additionally, it promotes transcription of mitochondrial transcription factor A, which then regulates mitochondrial DNA-encoded genes [19]. Furthermore, estrogen receptor β (ERβ) regulates the basal expression of superoxide dismutase 2 (SOD2) and plays a protective role in tissue damage [20–22]. Interestingly, ERβ expression can be suppressed by epigenetic changes, such as histone hypermethylation, which can be inherited by subsequent differentiated cells, thus triggering cellular dysfunction [23, 24]. Recently, it has been reported that ERβ promotes wound healing independent of its anti-inflammatory effect, although the precise mechanism remains unclear [25]. We hypothesize that diabetes-mediated oxidative stress may suppress ERβ and its target genes through epigenetic changes on the ERβ promoter, subsequently delaying wound healing.

Pterostilbene (trans-3, 5-dimethoxy-4′-hydroxystilbene; PTE) is a natural dimethylated derivative of resveratrol (RSV) that is primarily found in blueberries [26]. PTE has many pharmacological biological functions that are similar to that of RSV, including antioxidant, anti-inflammation, anti-tumor, immune modulation and anti-diabetic activities [27]. However, PTE also has more favorable pharmacokinetic properties, namely 2 methoxyl groups with higher lipophilicity compared to RSV, resulting in better bioavailability, longer half-life, lower toxicity and greater membrane permeability [28–30]. We have recently found that RSV can ameliorate oxidative stress-mediated epigenetic changes on the ERβ promoter through its potential antioxidant effects [17, 24], leading us to suppose that PTE may have an even stronger effect due to its higher bioavailability.

In this study, we aimed to investigate the potential effect of PTE and HSCs on diabetic wound healing. Our preliminary results showed that PTE exerts a stronger wound-healing effect in streptozocin (STZ)-induced diabetic rats, compared to RSV, because PTE treatment can ameliorate diabetes-induced epigenetic changes on the ERβ promoter in HSC, while RSV cannot. We then conducted further investigation using bone marrow transplantation (BMT) of PTE- or RSV-treated diabetic HSCs to rats with cutaneous burn injury. We found that BMT of PTE-treated HSCs can protect against diabetes-induced epigenetic changes on the ERβ promoter in both PBMCs and macrophages, subsequently reversing diabetes-induced oxidative stress, mitochondrial dysfunction and inflammation and eventually accelerating wound healing, while RSV treatment had little effect on diabetic HSCs. This study indicates that HSCs may play an important role in wound healing by transferring epigenetic modifications to subsequent PBMCs and macrophages during differentiation. Additionally, PTE can accelerate diabetic wound healing by ameliorating diabetes-induced epigenetic modifications in HSC, providing a novel therapeutic strategy for treating delayed diabetic wound healing through PTE-mediated activation in HSCs.

## Methods

A detailed description of the methods can be found in online supplementary material, and the primers used in this study are shown in Table S1. The animal protocol conformed to US National Institutes of Health guidelines (Guide for the Care and Use of Laboratory Animals, No.8523, revised 1996), and was reviewed and approved by the Institutional Animal Care and Use Committee from Wuhan University.

### Materials and reagents

Antibodies for β-actin (#sc-47 778), ERβ (#sc-137 381), NRF1 (#sc-101 102) and SOD2 (#sc-30 080) were obtained from Santa Cruz Biotechnology (Shanghai, China). The antibodies for CD31 (#ab24590), histone H3 lysine 9 dimethylation (H3K9me2) (#ab1220), histone H3 lysine 9 trimethylation (#ab8898), H3 lysine 27 dimethylation (#ab24684) and H3 lysine 27 trimethylation (H3K27me3) (#ab6002), H2AX (#ab20669) and phospho-Ser139 Histone H2A.X (#ab2893) were obtained from Abcam. Measurement of 3-nitrotyrosine was conducted using a 3-nitrotyrosine enzyme-linked immunosorbent assay (ELISA) kit (#ab116691, Abcam) as per the manufacturer’s instructions. Comet assay was conducted using a CometAssay™ kit (#TA800) from R&D Systems Inc., and 8-hydroxy-2′-deoxyguanosine formation was measured using an OxiSelect™ oxidative DNA damage ELISA kit (#STA320, Cell Biolabs Inc.) as per the manufacturer’s instructions. Protein concentration was measured using a Coomassie protein assay kit (Pierce Biotechnology). RSV (#R5010), STZ (#S0130) and PTE (#P1499) were obtained from Sigma (Shanghai, China).

### Preparation of green fluorescent protein (GFP) lentivirus particles for infection of HSCs

The pLenti-GFP Lentiviral Control Vector (#LTV-400) and related products were obtained from Cell Biolabs Inc. The lentiviral supernatant was produced by cotransfecting 293 T cells (#LTV-100) with the pLenti-GFP and ViraSafe™ Lentiviral Packaging System (#VPK-206). The lentivirus was concentrated and purified using the ViraBind™ Lentivirus Concentration and Purification Kit (#VPK-090), and the virus was used to infect isolated HSCs using the ViraDuctin™ Lentivirus Transduction Kit (#LTV-200).

### *In vivo* rat experiments

**Rat protocol 1: generation of diabetic rats** Chronic diabetic rats (2 months old) were induced by injection of 50 mg/kg STZ (0.05 M sodium citrate, pH 5.5) after an 8-hour fasting period. The blood glucose was monitored one week after injection—animals with blood glucose levels >300 mg/dl for 3 consecutive days were considered positive, while control (CTL) rats received only vehicle (VEH) injection [31, 32].

**Rat protocol 2: rat models of cutaneous burn** The diabetic rats from protocol 1 described above were subjected to a model of cutaneous burn injury after two weeks of STZ injection. The dorsum of each rat was shaved with electric clippers and depilated with Nair. Rats were anesthetized by inhalation of 5% isoflurane, and then the cutaneous burn injury was made on the dorsa of the rats by exposure to a hot copper pillar (2 cm in diameter) at 75°C for 15 seconds, and the subsequent wound-healing process was monitored and evaluated [31, 32].

**Rat protocol 3: treatments of rat models of cutaneous burns** The rats from protocol 2 received treatments of either VEH, RSV or PTE, which was first dissolved in 1% DMSO And diluted 10 times in 0.9% NaCl solution prior to intraperitoneal administration every 3 days at a dose of 15 mg/kg for 4 weeks starting from 1 week before the burn injury. The experimental rats were randomly separated into 4 groups as follows: group 1: CTL rats that received VEH treatment (CTL/VEH); group 2: STZ-induced diabetic rats that received VEH treatment (STZ/VEH); group 3: STZ-induced diabetic rats that received RSV treatment (STZ/RSV); and group 4: STZ-induced diabetic rats that received PTE treatment (STZ/PTE). During the treatment, the wound-healing process was monitored and evaluated. After treatment, the rats were sacrificed and HSCs were isolated from the tibia and femur for either BMT or biomedical analysis, including gene expression, chromatin immunoprecipitation analysis, SOD2 activity, oxidative stress, DNA damage and mitochondrial function. The PBMCs were also separated from blood using Ficoll-Paque Plus lymphocyte separation medium for further biomedical analysis.

**Rat protocol 4: BMT of HSCs** Male rats (2 months old) were used as recipients for BMT. The HSCs were isolated and characterized from rats in protocol 1 [33], purified by density centrifugation using Histopaque 1083® (#-1083–1, Sigma) and then resuspended in 10 ml of RPMI 1640 supplemented with 10% fetal bovine serum and 2 mM EDTA. The recipient male rats were lethally irradiated with 2 doses of 6 Gy 3 hours apart [34], and after 4 hours of irradiation, 2 × 10^6^ of isolated HSC cells Were systemically transplanted by tail-vein injection. All transplant-recipient rats were set aside for a minimum of 4 weeks to allow for complete reconstitution of the bone marrow [35] before they were then used for wound-healing analysis. The experimental rats were randomly separated into 4 groups as follows: rats with BMT of HSCs from CTL/VEH (BMT-CTL/VEH); rats with BMT of HSCs from STZ/VEH (BMT-STZ/VEH); rats with BMT of HSCs from STZ/RSV (BMT-STZ/RSV); and rats with BMT of HSCs from STZ/PTE (BMT-STZ/PTE). The rats with BMT transplantation of HSCs were subjected to a model of cutaneous burn injury for subsequent wound-healing analysis.

**Rat protocol 5: wound healing measurement** Digital photographs of the wounds were taken every 2 days for 21 days. Wound area was quantified as percentage of the original wound size using ImageJ software. At the indicated time points, wounds were excised and snap-frozen or, alternatively, processed for either H&E staining or immunohistochemistry (IHC). Images were taken using a Carl Zeiss MIRAX MIDI slide scanner and the analyses were performed using a 3DHISTECH Panoramic Viewer for the quantification of granulation tissue deposition [36]. Vascular density was detected on frozen sections by IHC using CD31 mouse monoclonal antibody. For quantification of CD31 positivity, wounds were analysed under ×200 magnification, and the number of positive 6 cells per high-power field were counted. All counts and observations were performed by a blinded observer [7]. Cytokine levels from rat serum were measured using ELISA kits (R&D Systems) and the peritoneal macrophages were isolated for gene expression [7, 31, 32].

### Isolation and characterization of HSCs

The HSC preparation procedure used was a minor modification of that previously described [13, 33]. In brief, whole bone marrow cells were collected from the tibias of treated rats. PBMCs were stained with antibodies for the identification of HSCs (c-Kit^+^/Sca1^+^/Lineage^−^), and the following antibodies were used: c-Kit-PE (#sc-365 504 PE, Santa Cruz Biotechnology), Sca-1-FITC, (react to rat, customized antibody from Dr Haimou Zhang, Hubei University) and an anti-lineage antibody cocktail, which was comprised of a mixture of PE-Cy5-conjugated antibodies, including anti-B220, anti-CD4, anti-CD8, anti-Gr-1, anti-Mac-1 and anti-TER119 (react to rat, customized antibodies from Dr Haimou Zhang, Hubei University). For HSC sorting, the debris and dead and clumped cells were removed to obtain single, viable cells, then the Sca-1-positive, c-Kit-positive and lineage-negative cell population were isolated by HSC sorting; the FACS analysis was performed using a BD FACSMelody™ Cell Sorter.

### Isolation of rat PBMCs

Heparinized peripheral blood, collected from rats by puncturing the heart, was diluted 1:3 with Hank’s balanced salts solution (HBSS) without Ca^2+^/Mg^2+^. The diluted blood was layered onto 15 ml of Ficoll-Paque in 50-ml sterile centrifuge tubes, followed by centrifugation at 300 × g at 20°C for 40 minutes. The PBMC layers were then harvested and washed 3 times by HBSS solution. The pellets were then resuspended with lysing buffer containing 150 mM NH_4_Cl, 1.0 mM KHCO_3_ and 0.1 mM Na_2_ EDTA (pH 7.4) and incubated for 5 minutes at room temperature to remove contaminated red cells. The cell suspensions were then centrifuged and washed twice with HBSS solution, then the cell pellet was resuspended for further biomedical analysis.

### Isolation of rat peritoneal macrophages

Macrophages were isolated from the peritoneal cavity of treated experimental rats. A 0.2 ml/ml solution of Concanavalin A was prepared in phosphate-buffered saline (PBS) and 1 ml was injected intraperitoneally into each rat. The rats were anesthetized using isoflurane 3 days after injection and a cardiac puncture was conducted to remove as much blood as possible. The abdominal skin was opened and 10 ml of warm PBS plus 1% of penicillin and streptomycin was injected intraperitoneally. After a gentle massage of the abdomen, a small incision was made in the abdominal wall to collect the fluid into a sterile 50-ml conical tube. The abdominal cavity was then rinsed twice with warm PBS plus 1% of penicillin and streptomycin and the collected fluid was centrifuged at 1000 rpm for 5 minutes. Sedimentary cells were resuspended with DMEM complete medium (containing 10% fetal bovine serum, 5 mM glucose, 100 U/ml penicillin and 100 g/ml streptomycin), adjusted to the required concentration and then incubated at 37°C in 5% CO_2_ for 6 hours. Adherent cells were collected and cultured for 18 hours, followed by subsequent analysis [37, 38].

### Wound macrophage isolation

Wound tissue was harvested on day 15 after burn injury by 6-mm punch biopsy as previously reported, but with minor modifications. In brief, the wound tissues were digested at 37°C for 30 minutes with 50 mg/ml Liberase (#5401020001, Sigma) and 20 units/ml DNase I (#D4263, Sigma). Samples were filtered over a 100-mm cell strainer to produce a single-cell suspension. Cells were then incubated with fluorescein isothiocyanate-labeled anti-CD3, anti-CD19 and anti-Ly6G (BioLegend), followed by anti-fluorescein isothiocyanate microbeads (Miltenyi Biotec). The flow-through was then incubated with anti-CD11b microbeads (Miltenyi Biotec) to isolate the non-neutrophil, non-lymphocyte, CD11b + cells. Cells were then used to count the GFP-positive cells under the fluorescence microscope [39].

### Immunostaining

The treated cells were transferred to cover slips and the cells were fixed in 4% paraformaldehyde for 20 minutes before being incubated with 0.3% Triton X-100 in PBS for 15 minutes. After blocking with 5% goat serum in PBS at room temperature for 30 minutes, cells were incubated with 8-oxo-dG Anti-mouse antibody (# 4354-MC-050, from Novus Biologicals) for 12 hours at 4°C and subsequently with secondary antibody Alexa Fluor 488. The cover slips were then mounted by antifade Mountant with DAPI (staining nuclei, in blue). The photographs were taken using a Confocal Laser Microscope (Leica, 20x lens) and quantitated by Image J. software [17].

### ELISA

Rat interleukins from either supernatant or serum, including interleukin-1β (IL1β), interleukin-6 (IL6) and monocyte chemoattractant protein-1 (MCP1), were measured by rat IL-1β/IL-1F2 Quantikine ELISA kit (#RLB00), rat IL-6 Quantikine ELISA kit (#RRA00) and rat JE/MCP-1/CCL2 DuoSet ELISA kit (#DY3144–05), respectively, according to the manufacturer’s instructions (R&D Systems) [40].

### Immunohistochemistry

The tissues were dissected and snap-frozen in the OCT compound. The 10-μm sections were cut by clean microtome, mounted on PEN-membrane slides (2.0 μm, Leica) and stored at −20°C before use. The slides were first fixed by 3.7% formaldehyde at 37°C for 15 minutes, permeabilized by 1% BSA and 0.2% Triton X-100 in PBS for 1 hour, and then blotted with 40 μg/ml (diluted 1:20) of either MCP1 or CD31 mouse monoclonal antibody for 2 hours. They were then washed 3 times and the Texas-red (for CD31) or DAB (for MCP1) labeled anti-mouse secondary antibody (1:200) was added for blotting for another 1 hour. After thorough washing, the slides were visualized and photographed. The relative densities of each group were quantitated for protein expression using ImageJ software [31, 32, 41].

**Figure 1.**
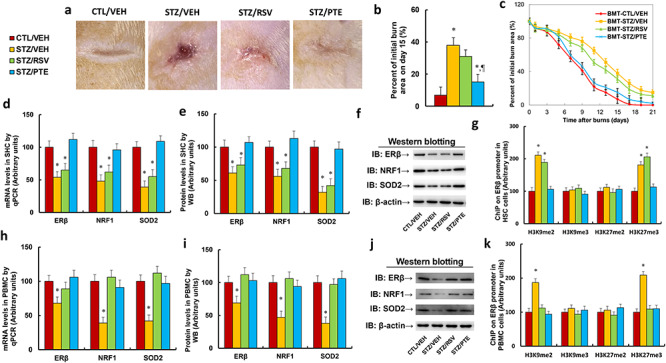
PTE has a stronger effect than RSV on accelerating diabetic wound healing by ameliorating diabetes-induced epigenetic modifications on the ERβ promoter in HSCs. Experimental rats with burn injuries were divided into 4 groups: control rats (CTL/VEH), STZ diabetic rats (STZ/VEH), STZ rats treated with 15 mg/kg/day of RSV (STZ/RSV) and STZ rats treated with 15 mg/kg body weight of PTE. **(a)** Photographs of representative wounds on day 15 post-burn. **(b)** Quantitation of burn area on day 15 post burn, n = 8. **(c)** Graphical depiction of wound areas on different days post-burn, n = 8. **(d–g)** HSCs were collected from treated rats for biomedical analysis. **(d)** mRNA levels determined by qPCR, n = 4. **(e)** Protein quantitation by WB for **(f)**, n = 5. **(f)** Representative picture for western blots. **(g)** ChIP analysis on ERβ promoter, n = 4. **(h–k)** PBMCs were collected from treated rats for biomedical analysis. **(h)** mRNA levels by qPCR, n = 4. **(i)** Protein quantitation by WB for (j), n = 5. **(j)** Representative picture for western blots. **(k)** ChIP analysis on ERβ promoter, n = 4. For bars in graphs marked with an asterisk, *p* < 0.05 *vs* CTL/VEH group; for paragraph marks, *p* < 0.05 *vs* STZ/RSV group. Data are expressed as mean ± SEM. *PTE* pterostilbene, *RSV* resveratrol, *ERβ* estrogen receptor β, *HSCs* hematopoietic stem cells, *CTL* control, *VEH* vehicle, *STZ* streptozotocin, *PBMCs* peripheral blood mononuclear cells, *NRF1* nuclear respiratory factor-1, *SOD2* superoxide dismutase 2, *H3K9me2* histone H3 lysine 9 dimethylation, *H3K9me3* histone H3 lysine 9 trimethylation, *H3K27me2* H3 lysine 27 dimethylation, *H3K27me3* H3 lysine 27 trimethylation

## Results

### PTE has a stronger effect than RSV in accelerating diabetic wound healing because PTE can ameliorate diabetes-induced epigenetic modifications on ERβ promoter in HSCs, while RSV cannot

We first evaluated the effect of PTE and RSV on diabetic wound healing in rats. The experimental rats with burn injuries were divided into 4 groups: control rats (CTL/VEH), STZ diabetic rats (STZ/VEH), STZ rats treated with 15 mg/kg/day of RSV (STZ/RSV) or STZ rats that received 15 mg/kg of PTE (STZ/PTE). The results showed that diabetes (STZ/VEH) significantly delayed wound healing and that RSV treatment (STZ/RSV) slightly, and PTE significantly, accelerated wound healing compared to the control (CTL/VEH) group (Figure 1a, b, c). We then evaluated the potential effect of RSV and PTE on HSCs (Figure 1d, e, f, g). The results showed that STZ/VEH treatment decreased mRNA expression of ERβ, NRF1 and SOD2 to 54%, 48% and 39%, respectively, compared to the CTL/VEH group. RSV treatment (STZ/RSV) had little effect, while PTE treatment (STZ/PTE) completely restored STZ-induced gene suppression (Figure 1d). Furthermore, we measured the protein levels for those genes and noticed an expression pattern similar to that of the mRNA (Figure 1e, f and Figure S1a). We then measured the epigenetic changes on the ERβ promoter (Figure 1g). The results showed that STZ/VEH treatment increased H3K9me2 and H3K27me3 modification to 211% and 181%, respectively, compared to the CTL/VEH group. Again, RSV had no effect, while PTE completely restored STZ/VEH-mediated epigenetic changes. We finally evaluated the effect of RSV and PTE on PBMCs (Figure 1h, i, j, k). The results showed that STZ/VEH treatment decreased mRNA expression of ERβ, NRF1 and SOD2 to 68%, 39% and 42%, respectively, compared to the CTL/VEH group, while both RSV (STZ/RSV) and PTE (STZ/PTE) treatment completely restored diabetes-induced (STZ/VEH) gene suppression (Figure 1h). We then measured the protein levels for those genes and found an expression pattern similar to that of the mRNA (Figure 1i, j and Figure S1b). We then measured the epigenetic changes on the ERβ promoter (Figure 1k). The results showed that STZ/VEH treatment increased H3K9me2 and H3K27me3 modification to 187% and 209%, respectively, compared to the CTL/VEH group. Again, both RSV and PTE completely reversed STZ/VEH-mediated epigenetic changes. Our results showed that PTE has stronger effect than RSV on the acceleration of diabetic wound healing; additionally, PTE can protect against diabetes-induced epigenetic changes on the ERβ promoter in both HSCs and PBMCs, while RSV has an effect in PBMCs, but not in HSCs. This indicates that PTE may contribute to accelerated wound healing via epigenetic modifications in HSCs.

**Figure 2.**
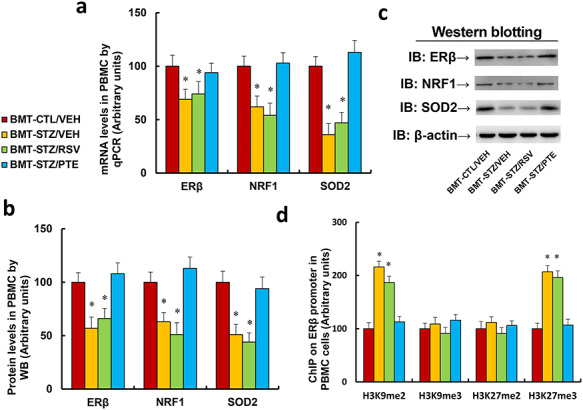
BMT of PTE-treated diabetic HSCs protects against diabetes-induced epigenetic modifications on the ERβ promoter in PBMCs. Experimental rats were randomly separated into 4 groups as follows: rats with BMT of HSCs from CTL/VEH (BMT-CTL/VEH); rats with BMT of HSCs from STZ/VEH (BMT-STZ/VEH); rats with BMT of HSCs from STZ/RSV (BMT-STZ/RSV); and rats with BMT of HSCs from STZ/PTE (BMT-STZ/PTE). The rats were subjected to a model of cutaneous burn injury and PBMCs were collected for biomedical analysis after a 3-week period post-burn. **(a)** mRNA levels by qPCR, n = 4. **(b)** Protein quantitation by WB for (c), n = 5. **(c)** Representative picture for western blots. **(d)** ChIP analysis on ERβ promoter, n = 4. For bars in graphs marked with an asterisk, *p* < 0.05 vs BMT-CTL/VEH group. Data are expressed as mean ± SEM. *BMT* bone marrow transplantation, *PTE* pterostilbene, *HSCs* hematopoietic stem cells, *ERβ* estrogen receptor β, *PBMCs* peripheral blood mononuclear cells, *CTL* control, *VEH* vehicle, *STZ* streptozotocin, *RSV* resveratrol, *NRF1* nuclear respiratory factor-1, *SOD2* superoxide dismutase 2, *H3K9me2* histone H3 lysine 9 dimethylation, *H3K9me3* histone H3 lysine 9 trimethylation, *H3K27me2* H3 lysine 27 dimethylation, *H3K27me3* H3 lysine 27 trimethylation

### Bone marrow transplantation of PTE-treated diabetic HSCs protects against diabetes-induced epigenetic modifications on the ERβ promoter in PBMCs

The experimental rats were randomly separated into 4 groups as follows: rats with BMT of HSCs from CTL/VEH (BMT-CTL/VEH); rats with BMT of HSCs from STZ/VEH (BMT-STZ/VEH); rats with BMT of HSCs from STZ/RSV (BMT-STZ/RSV); and rats with BMT of HSCs from STZ/PTE (BMT-STZ/PTE). The rats were subjected to a model of cutaneous burn injury and PBMCs were collected for biomedical analysis after a 3-week wound-healing period. We first evaluated mRNA expression levels. The results showed that BMT-STZ/VEH treatment decreased mRNA expression of ERβ, NRF1 and SOD2 to 69%, 62% and 36%, respectively, compared to the BMT-CTL/VEH group; RSV treatment (BMT-STZ/RSV) had little effect, while PTE treatment (BMT-STZ/PTE) completely restored BMT-STZ/VEH treatment-induced gene suppression (Figure 2a). We then measured the protein levels for those genes and identified an expression pattern similar to that of the mRNA (Figure 2b, c and Figure S1c). We then measured the epigenetic changes on the ERβ promoter (Figure 2d). The results showed that BMT-STZ/VEH treatment increased H3K9me2 and H3K27me3 modification to 216% and 207%, respectively, compared to the BMT-CTL/VEH group. Again, BMT-STZ/RSV treatment had no effect, while BMT-STZ/PTE completely reversed BMT-STZ/VEH-mediated epigenetic changes. Our results indicate that BMT of PTE-treated diabetic HSCs protects against diabetes-induced epigenetic modifications on the ERβ promoter in PBMCs.

### Bone marrow transplantation of PTE-treated diabetic HSCs protects against diabetes-induced oxidative stress in PBMCs

**Figure 3.**
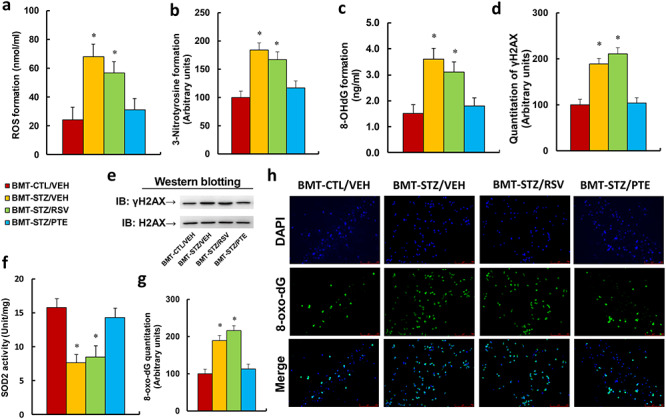
BMT of PTE-treated diabetic HSCs protects against diabetes-induced oxidative stress in PBMCs. Experimental rats were randomly separated into 4 groups as follows: rats with BMT of HSCs from CTL/VEH (BMT-CTL/VEH); rats with BMT of HSCs from STZ/VEH (BMT-STZ/VEH); rats with BMT of HSCs from STZ/RSV (BMT-STZ/RSV); and rats with BMT of HSCs from STZ/PTE (BMT-STZ/PTE). The rats were subjected to a model of cutaneous burn injury and PBMCs were collected for biomedical analysis after a 3-week period post-burn. **(a)** ROS formation in PBMCs, n = 5. **(b)** Quantitation of 3-nitrotyrosine formation, n = 5. **(c)** 8-OHdG formation, n = 5. **(d)** Quantitation of γH2AX formation. **(e)** Representative γH2AX western blotting band for (d), n = 5. **(f)** SOD2 activity, n = 5. **(g)** Quantitation of 8-oxo-dG formation, n = 5. **(h)** Representative pictures of 8-oxo-dG staining for oxidative stress (green) and DAPI staining for nuclei (blue) in PBMC, n = 4. For bars in graphs marked with an asterisk, *p* < 0.05 *vs* BMT-CTL/VEH group. Data are expressed as mean ± SEM. *BMT* bone marrow transplantation, *PTE* pterostilbene, *HSCs* hematopoietic stem cells, *PBMCs* peripheral blood mononuclear cells, *CTL* control, *VEH* vehicle, *STZ* streptozotocin, *RSV* resveratrol, *ROS* reactive oxygen species, *OHdG* 8-hydroxy-2′-deoxyguanosine, *γH2AX* phospho-Ser139 histone H2A.X, *SOD2* superoxide dismutase 2, *DAPI* 4,6-diamidino-2-phenylindole, *H2AX* H2A.X Variant Histone, *8-oxo-dG* 8-Oxo-2′-deoxyguanosine

We evaluated the potential effect of BMT on diabetes-induced oxidative stress. The results showed that BMT-STZ/VEH treatment increased reactive oxygen species (Figure 3a) and 3-nitrotyrosine formation (Figure 3b) to 283% and 184%, respectively, compared to the BMT-CTL/VEH group. Furthermore, it increased 8-hydroxy-2′-deoxyguanosine formation (Figure 3c) and phospho-Ser139 histone H2A.X formation (Figure 3d, e and Figure S1d) to 240% and 189%, respectively. In addition, BMT-STZ/VEH treatment decreased SOD2 activity (Figure 3f) to 48% and increased 8-oxo-dG formation (Figure 3g, h) to 189% compared to the BMT-CTL/VEH group. Again, BMT-STZ/RSV treatment had no effect, while BMT-STZ/PTE completely reversed BMT-STZ/VEH-mediated oxidative stress. Our results indicate that BMT of PTE-treated diabetic HSCs protects against diabetes-induced oxidative stress in PBMCs.

### Bone marrow transplantation of PTE-treated diabetic HSCs protects against diabetes-induced mitochondrial dysfunction in PBMCs

**Figure 4.**
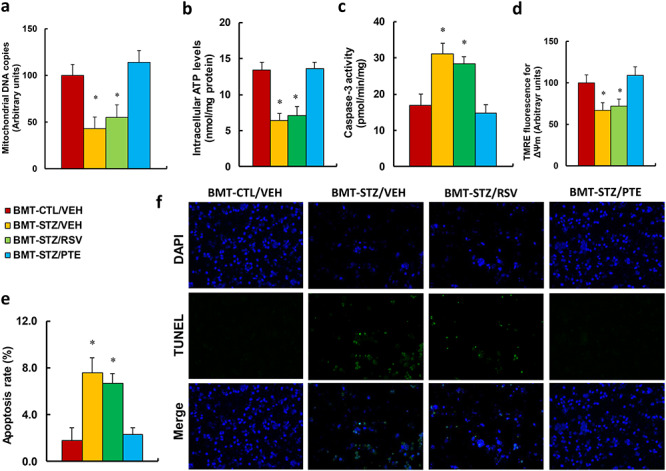
BMT of PTE-treated diabetic HSCs protects against diabetes-induced mitochondrial dysfunction in PBMCs. Experimental rats were randomly separated into 4 groups as follows: rats with BMT of HSCs from CTL/VEH (BMT-CTL/VEH); rats with BMT of HSCs from STZ/VEH (BMT-STZ/VEH); rats with BMT of HSCs from STZ/RSV (BMT-STZ/RSV); and rats with BMT of HSCs from STZ/PTE (BMT-STZ/PTE). The rats were subjected to a model of cutaneous burn injury and the PBMCs were collected for biomedical analysis after a 3-week period post-burn. **(a)** mDNA copies, n = 4; **(b)** the intracellular ATP level, n = 4. **(c)** Caspase-3 activity, n = 5. **(d)** ∆ᴪm by TMRE fluorescence, n = 5. **(e)** Apoptosis rate by TUNEL assay, n = 5. **(f)** Representative pictures for (e). For bars in graphs marked with an asterisk, *p* < 0.05 *vs* BMT-CTL/VEH group. Data are expressed as mean ± SEM. *BMT* bone marrow transplantation, *PTE* pterostilbene, *HSCs* hematopoietic stem cells, *PBMCs* peripheral blood mononuclear cells, *CTL* control, *VEH* vehicle, *STZ* streptozotocin, *RSV* resveratrol, *ATP* Adenosine triphosphate, *TMRE* tetramethyl rhodamine ethyl ester, *TUNEL* terminal deoxynucleotidyl transferase dUTP nick end labeling

We evaluated the potential effect of BMT on diabetes-induced mitochondrial dysfunction. The results showed that BMT-STZ/VEH treatment decreased mitochondrial DNA copies (Figure 4a) and intracellular ATP levels (Figure 4b) to 43% and 48%, respectively, compared to the BMT-CTL/VEH group. Furthermore, it increased caspase-3 activity (Figure 4c) to 185%, and decreased mitochondrial membrane potential (Figure 4d) to 67%. In addition, BMT-STZ/VEH treatment increased the apoptosis rate (Figure 4e, f) to 422% compared to the BMT-CTL/VEH group. Again, BMT-STZ/RSV treatment had no effect, while BMT-STZ/PTE completely reversed BMT-STZ/VEH-mediated mitochondrial dysfunction. Our results indicate that BMT of PTE-treated diabetic HSCs protects against diabetes-induced mitochondrial dysfunction in PBMCs.

### Bone marrow transplantation of PTE-treated diabetic HSC protects against diabetes-induced epigenetic changes on the ERβ promoter and pro-inflammatory cytokine secretion in macrophages

**Figure 5.**
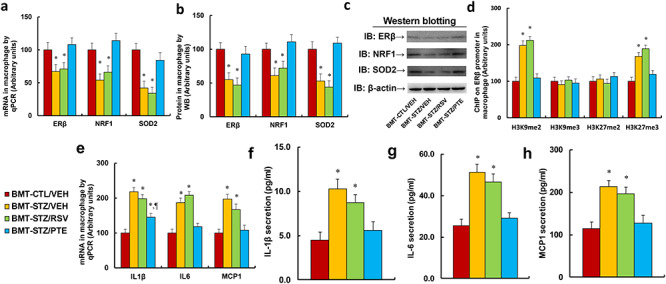
BMT of PTE-treated diabetic HSCs protects against diabetes-induced epigenetic changes on ERβ promoter and pro-inflammatory cytokine secretion in macrophages. Experimental rats were randomly separated into 4 groups as follows: rats with BMT of HSCs from CTL/VEH (BMT-CTL/VEH); rats with BMT of HSCs from STZ/VEH (BMT-STZ/VEH); rats with BMT of HSCs from STZ/RSV (BMT-STZ/RSV); and rats with BMT of HSCs from STZ/PTE (BMT-STZ/PTE). The rats were subjected to a model of cutaneous burn injury and the macrophages were collected for biomedical analysis after a 3-week period post-burn. **(a)** mRNA levels by qPCR, n = 4. **(b)** Protein quantitation by WB for **(c)**, n = 5. **(c)** Representative picture for western blots. **(d)** ChIP analysis on ERβ promoter, n = 4. **(e)** mRNA levels for pro-inflammatory cytokines, n = 4. **(f)** IL1β secretion, n = 5. **(g)** IL6 secretion, n = 5. **(h)** MCP1 secretion, n = 5. For bars in graphs marked with an asterisk, *p* < 0.05 *vs* BMT-CTL/VEH group; for paragraph marks, *p* < 0.05 *vs* BMT-STZ/RSV group. Data are expressed as mean ± SEM. *BMT* bone marrow transplantation, *PTE* pterostilbene, *HSCs* hematopoietic stem cells, *ERβ* estrogen receptor β, *CTL* control, *VEH* vehicle, *STZ* streptozotocin, *RSV* resveratrol, *IL1β* interleukin-1β, *IL6* interleukin-6, *MCP1* monocyte chemoattractant protein-1, *NRF1* nuclear respiratory factor-1, *SOD2* superoxide dismutase 2, *H3K9me2* histone H3 lysine 9 dimethylation, *H3K9me3* histone H3 lysine 9 trimethylation, *H3K27me2* H3 lysine 27 dimethylation, *H3K27me3* H3 lysine 27 trimethylation, *ChIP* ChromatinImmunoprecipitation, *WB* western blotting, *H&E* Hematoxylin and eosin

The above BMT-treated rats were subjected to a model of cutaneous burn injury, and the macrophages were collected for biomedical analysis after a 3-week wound healing period. We first evaluated the mRNA expression in macrophages. The results showed that BMT-STZ/VEH treatment decreased mRNA expression of ERβ, NRF1 and SOD2 to 67%, 54% and 42%, respectively, compared to the BMT-CTL/VEH group. BMT-STZ/RSV treatment had little effect, while BMT-STZ/PTE treatment completely reversed BMT-STZ/VEH treatment-induced gene suppression (Figure 5a). We then measured the protein levels for those genes and identified an expression pattern similar to that of the mRNA (Figure 5b, c and Figure S1e). We then measured the epigenetic changes on the ERβ promoter (Figure 5d). The results showed that BMT-STZ/VEH treatment increased H3K9me2 and H3K27me3 modification to 198% and 168%, respectively, compared to the BMT-CTL/VEH group. Again, BMT-STZ/RSV treatment had no effect, while BMT-STZ/PTE completely reversed BMT-STZ/VEH-mediated epigenetic changes. We then measured the gene expression for the pro-inflammatory cytokines. The results showed that BMT-STZ/VEH treatment increased mRNA expression of IL1β, IL6 and MCP1 to 218%, 187% and 197%, respectively, compared to the BMT-CTL/VEH group; BMT-STZ/RSV treatment had little effect, while BMT-STZ/PTE treatment reversed BMT-STZ/VEH treatment-induced cytokine expression partially for IL1β and completely for IL6 and MCP1 (Figure 5e). We then measured mRNA expression for the cytokine levels in macrophages. The results showed that BMT-STZ/VEH treatment increased cytokine secretion of IL1β (Figure 5f), IL6 (Figure 5g) and MCP1 (Figure 5h) to 229%, 200% and 187%, respectively, compared to the BMT-CTL/VEH group. Again, BMT-STZ/RSV treatment had no effect, while BMT-STZ/PTE completely reversed BMT-STZ/VEH-mediated cytokine secretion in macrophages. Our data indicate that BMT of PTE-treated diabetic HSCs protects against diabetes-induced epigenetic changes on the ERβ promoter and pro-inflammatory cytokine secretion in macrophages.

**Figure 6.**
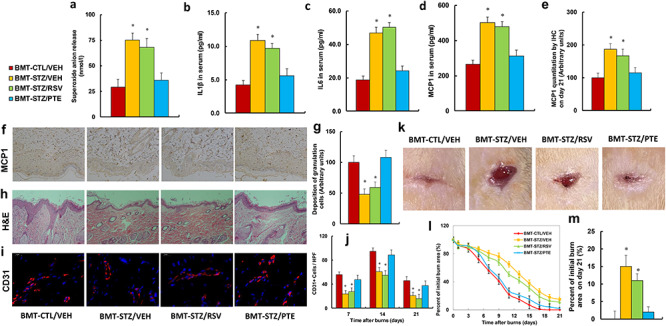
BMT of PTE-treated diabetic HSCs accelerates diabetic wound healing by ameliorating diabetes-induced oxidative stress and inflammation. Experimental rats were randomly separated into 4 groups as follows: rats with BMT of HSCs from CTL/VEH (BMT-CTL/VEH); rats with BMT of HSCs from STZ/VEH (BMT-STZ/VEH); rats with BMT of HSCs from STZ/RSV (BMT-STZ/RSV); and rats with BMT of HSCs from STZ/PTE (BMT-STZ/PTE). The rats were subjected to a model of cutaneous burn injury and the wound-healing process was evaluated, followed by biomedical analysis. **(a)** Superoxide anion release from wound tissues, n = 5. **(b)** IL1β in serum, n = 5. **(c)** IL6 in serum, n = 5. **(d)** MCP1 in serum, n = 5. **(e)** MCP1 quantitation by IHC from wound tissues on day 21, n = 5. **(f)** Representative pictures for (e). **(g)** Deposition of granulation cells from (h), n = 8. **(h)** H&E staining of wound tissues on day 21 after burn injury with occurrence of granulation cells in the wounds. **(i)** Representative pictures for evaluation of vascularity (assessed by CD31 IHC) for (j). **(j)** Numbers of CD31-positive vessels per high-power field on day 21 after burn injury, n = 8. **(k)** Photographs of representative wounds on day 21 after burn injury. **(l)** Graphical depiction of wound areas on different days after burn injury, n = 8. **(m)** Quantitation of burn area on day 21, n = 8. For bars in graphs marked with an asterisk, *p* < 0.05 *vs* CTL/VEH group. Data are expressed as mean ± SEM. *BMT* bone marrow transplantation, *PTE* pterostilbene, *HSCs* hematopoietic stem cells, *CTL* control, *VEH* vehicle, *STZ* streptozotocin, *RSV* resveratrol, *IL1β* interleukin-1β, *IL6* interleukin-6, *MCP1* monocyte chemoattractant protein-1, *IHC* immunohistochemistry

**Figure 7.**
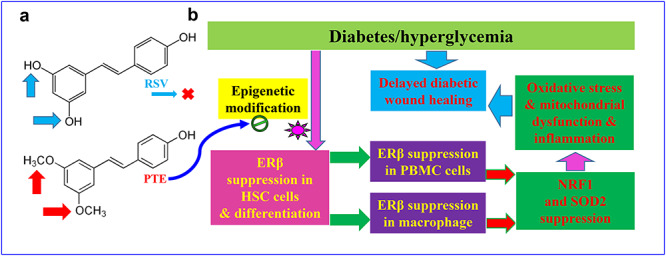
Schematic model for PTE-mediated wound healing by modulating diabetes-induced ERβ suppression in HSCs. **(a)** Comparison of molecular structure between RSV and PTE. **(b)** Schematic model for PTE-mediated diabetic wound healing. *PTE* pterostilbene, *ERβ* estrogen receptor β, *HSCs* hematopoietic stem cells, *RSV* resveratrol, *PBMCs* peripheral blood mononuclear cells, *NRF1* nuclear respiratory factor-1, *SOD2* superoxide dismutase 2

### GFP lentivirus-infected HSCs that undergo bone marrow transplantation can differentiate into PBMCs and macrophages

Our results showed that diabetes-mediated epigenetic changes not only occurred in HSC cells, but also happened in PBMCs and macrophages. We therefore supposed that diabetes-mediated epigenetic changes in HSCs were inherited by PBMCs and macrophages during differentiation. The HSCs from donor rats were collected and infected by GFP lentivirus before BMT to recipient rats, and the GFP-positive cell rate was counted. After 4 weeks of BMT, the PBMCs and wound macrophages were isolated and the GFP-positive cells were again counted. The results showed that around 89% of HSCs were infected by GFP lentivirus before BMT, while after BMT, only 15.9% and 11.8% of PBMCs and macrophages, respectively, were found to be GFP-positive (Figure S2a, b). Our results indicate that HSCs can differentiate into PBMCs and wound macrophages after BMT.

### Bone marrow transplantation of PTE-treated diabetic HSCs accelerates diabetic wound healing by ameliorating diabetes-induced oxidative stress and inflammation

The above BMT-treated rats were subjected to a model of cutaneous burn injury before the biomedical parameters were analysed and the wound-healing process was evaluated. The results showed that BMT-STZ/VEH treatment increased superoxide anion release from local wound tissues to 259% (Figure 6a) and increased serum cytokine levels of IL1β (Figure 6b), IL6 (Figure 6c) and MCP1 (Figure 6d) to 260%, 250% and 191%, respectively, compared to the BMT-CTL/VEH group. Conversely, BMT treatment did not have any effect on the gene expression of ERβ and its target genes NRF1 and SOD2 from local tissues (Figure S3). We also evaluated the effect of BMT on local wound tissues using IHC or H&E staining techniques. The results showed that BMT-STZ/VEH treatment increased local MCP protein expression in brown/black color (on day 21) to 1871% (Figure 6e, f) and decreased deposition of granulation cells in the wounds (on day 21) to 48% (Figure 6g, h). We also evaluated neovascularization through IHC staining of CD31-positive cells (Figure 6i, j) and found that BMT-STZ/VEH treatment decreased the number of CD31-positive cells to 43%, 64% and 46% on days 7, 14 and 21, respectively, compared to the BMT-CTL/VEH group. Additionally, BMT-STZ/RSV treatment had no effect, while BMT-STZ/PTE treatment completely restored the BMT-STZ/VEH-mediated effect for all the above parameters. Finally, we measured the wound-healing rate at different time points with different BMT treatments. The results showed that transplantation of diabetic HSCs (BMT-STZ/VEH) significantly delayed wound healing compared to the BMT-CTL/VEH group, and BMT-STZ/RSV treatment slightly, while BMT-STZ/PTE treatment significantly, accelerated wound healing (Figure 6k, l, m). Our results indicate that BMT of PTE-treated diabetic HSCs accelerates burn wound healing by ameliorating diabetes-induced oxidative stress and inflammation.

### Schematic model for PTE-mediated wound healing by modulating diabetes-induced ERβ suppression in HSCs

We established a schematic model for PTE-mediated diabetic wound healing through epigenetic modifications in HSCs. PTE and RSV have similar molecular structures, while PTE has higher membrane permeability since it has 2 methoxyl groups compared to the 2 hydroxyl groups in RSV—this makes PTE has better bioavailability to reach HSC cells (Figure 7a). In diabetic conditions, ERβ expression is suppressed in HSCs due to diabetes-mediated epigenetic changes on the ERβ promoter, and the epigenetic modifications are inherited during differentiation of HSCs, resulting in subsequent ERβ suppression in both PBMCs and macrophages. ERβ suppression then inhibits the expression of its target genes, including NRF1 and SOD2, resulting in mitochondrial dysfunction, oxidative stress and inflammation in wound tissues, eventually leading to delayed diabetic wound healing. PTE can accelerate diabetic wound healing by rescuing diabetes-mediated epigenetic changes in HSCs (Figure 7b).

## Discussion

In this study, we demonstrated that PTE has a greater effect on the speed of diabetic wound healing than RSV by ameliorating diabetes-induced epigenetic changes on the ERβ promoter in HSCs. BMT of PTE-treated HSCs protects against diabetes-induced oxidative stress, mitochondrial dysfunction and inflammation by reversing dysfunction of PBMCs and macrophages, thereby accelerating cutaneous wound healing.

### Diabetes-mediated ERβ suppression in HSCs

Our results showed that STZ-induced diabetes induces histone methylation on the ERβ promoter and subsequently suppresses ERβ and its target genes, including NRF1 and SOD2, in HSCs. NRF1 regulates mitochondrial function through mitochondrial transcription factor A and PGC1α [18, 19], and the antioxidant enzyme SOD2 catalyses dismutation of mitochondrial superoxide anions. Epigenetic modification-mediated ERβ suppression in HSCs may result in consistent oxidative stress and mitochondrial dysfunction, which transfers to subsequent PBMCs and macrophages during differentiation and contributes to delayed diabetic wound healing.

### Role of HSCs in diabetic wound healing

Our results showed that STZ-mediated diabetes induces epigenetic changes, with suppression of ERβ and its target genes in HSCs. These properties are transferred to PBMCs and macrophages through differentiation, with subsequent intracellular consequences, including over-generation of reactive oxygen species, DNA damage, mitochondrial dysfunction and elevated pro-inflammatory cytokine release. BMT of these diabetes-treated HSCs delayed cutaneous wound healing in rats, indicating that HSCs may play an important role in diabetic wound healing [13]. In this study, we showed that BMT of PTE-treated diabetic HSCs reverses gene expression and epigenetic changes on the ERβ promoter in both PBMCs and macrophages, subsequently increasing expression of ERβ and its target genes while reducing oxidative stress, restoring mitochondrial dysfunction in PBMCs and normalizing elevated pro-inflammatory cytokines in macrophages. Reduced oxidative stress, mitochondrial dysfunction and pro-inflammatory cytokines may be the causative mechanisms for the acceleration of diabetic wound healing. Since it has already been shown that both PBMCs and macrophages mainly come from HSC differentiation [13, 42], and our data has shown that BMT of PTE-treated diabetic HSCs reverses epigenetic changes in both PBMCs and macrophages, we conclude that the epigenetic modifications in both PBMCs and macrophages are most likely inherited from HSCs during differentiation. In addition, we have shown that BMT of PTE-treated diabetic HSCs accelerates diabetic wound healing with recruited granulation cells. In order to further prove that the PBMCs and macrophages can inherit the same epigenetic modifications from HSCs, we conducted an additional experiment. The GFP lentivirus-infected HSCs (isolated from donor rats) were used for BMT, and isolated PBMCs and macrophages from recipient rats were found to have around 15.9% and 11.8% of GFP-positive cells, respectively (Figure S2). This further indicates that HSCs from donor rats can differentiate into PBMCs and wound macrophages in recipient rats after BMT; although the positivity rate is relatively low, this can be partly explained by partial removal of GFP-positive cells by the immune system of the recipient rats after BMT. The HSCs used for BMT as diabetic wound healing treatment should have had a higher survival rate, allowing more to reach the wound site, as they cells had no GFP interference.

### Role of PTE and RSV in diabetic wound healing

Our results show that PTE treatment protects against diabetes-induced epigenetic modifications on the ERβ promoter and subsequent suppression of ERβ and its target genes in both HSCs and PBMCs. RSV treatment showed a significant effect on PBMCs, but not HSCs, indicating that either RSV may not be able to reach HSCs or that levels cannot reach sufficient concentrations to reverse diabetes-induced epigenetic modifications in HSCs that are located in the bone marrow niche. This can be explained by the reasoning that PTE has much better bioavailability and membrane permeability as compared to RSV due to its 2 methoxyl groups [26–28]. In addition, we showed that PTE protects against diabetes-induced oxidative stress, mitochondria dysfunction and elevated cytokine release by ameliorating epigenetic changes on the ERβ promoter [17, 24], which subsequently accelerates cutaneous wound healing. Furthermore, our results show that PTE can significantly accelerate diabetic wound healing, while RSV has less effect, indicating that PTE may be a potential therapeutic strategy for diabetic wound healing.

## Conclusions

PTE has much stronger effect than RSV in accelerating diabetic wound healing because PTE can ameliorate diabetes-induced epigenetic modifications in HSCs, while RSV cannot. BMT of PTE-treated diabetic HSCs accelerates cutaneous wound healing by ameliorating diabetes-induced oxidative stress, mitochondria dysfunction and elevated pro-inflammatory cytokines in PBMCs and macrophages. We conclude that HSCs play an important role in wound healing and PTE may be a novel therapeutic strategy for treatment of diabetic wound healing.

## Supplementary Material

PTE-201030-SI_tkaa045

## Abbreviations

BMT: bone marrow transplantation; ELISA: enzyme-linked immunosorbent assay; ERβ: estrogen receptor β; H3K27me3: H3 lysine 27 trimethylation; H3K9me2: Histone H3 lysine 9 dimethylation; HBSS: Hank’s balanced salts solution; HSCs: hematopoietic stem cells; IHC: immunohistochemistry; IL1β: interleukin-1β; IL6: interleukin-6; MCP1: monocyte chemoattractant protein-1; NRF1: nuclear respiratory factor-1; PBMCs: peripheral blood mononuclear cells; PBS: phosphate-buffered saline; PTE: pterostilbene; ROS: reactive oxygen species; RSV: resveratrol; SOD2: superoxide dismutase 2; STZ: streptozotocin
