# Supplementary material for: Pterostilbene accelerates wound healing by modulating diabetes-induced estrogen receptor β suppression in hematopoietic stem cells
Source: Burns Trauma. 2021 Feb 22;9:tkaa045. doi: 10.1093/burnst/tkaa045 (PMC7901710; doi:10.1093/burnst/tkaa045)
Supplement: PTE-201030-SI_tkaa045 [file pte-201030-si_tkaa045.docx]

**Pterostilbene Accelerates Wound Healing by Modulating Diabetes-Induced ERβ Suppression in Hematopoietic Stem Cells**

Weiguo Xie^1^, Xueqing Zhou^1^, Weigang Hu^1^, Zhigang Chu^1^, Qiongfang Ruan^1^, Haimou Zhang^2^,

Min Li^1^, Hongyu Zhang^3^, Xiaodong Huang^1^, Paul Yao^1,#^

**Supplemental Information**

**Data S1: Materials and Methods**

Materials and Reagents. Antibodies for β-actin (sc-47778), ERβ (sc-137381), NRF1 (sc-101102) and SOD2 (sc-30080) were obtained from Santa Cruz Biotechnology (Shanghai, China). The antibodies for CD31 (ab24590), Histone H3 lysine 9 dimethylation (H3K9me2, #ab1220), Histone H3 lysine 9 trimethylation (H3K9me3, #ab8898), H3 lysine 27 dimethylation (H3K27me2, #ab24684) and H3 lysine 27 trimethylation (H3K27me3, #ab6002), H2AX (ab20669) and Phospho-Ser139 Histone H2A.X (γH2AX, #ab2893) were obtained from Abcam, and 3-nitrotyrosine (3-NT) was measured using the 3-Nitrotyrosine ELISA Kit (ab116691 from Abcam) per manufacturers’ instructions. Comet assay was conducted using a CometAssay™ kit (Cat No. TA800) from R&D Systems Inc, and the 8-hydroxy-2' -deoxyguanosine (8-OHdG) formation was measured using an OxiSelect™ Oxidative DNA Damage ELISA Kit (Cat No. STA320, from Cell Biolabs Inc.) according to manufacturers’ instructions. Protein concentration was measured using the Coomassie Protein Assay Kit (Pierce Biotechnology). The resveratrol (RSV, #R5010), streptozocin (STZ, #S0130) and pterostilbene (PTE, #P1499) were obtained from Sigma (Shanghai, China).

**Preparation of GFP lentivirus particles for HSC infection.** The pLenti-GFP Lentiviral Control Vector (#LTV-400) and the related product were obtained from Cell Biolabs Inc. The lentiviral supernatant was produced by cotransfecting 293T cells (#LTV-100) with pLenti-GFP and ViraSafe™ Lentiviral Packaging System (#VPK-206). The lentivirus was concentrated and purified using ViraBind™ Lentivirus Concentration and Purification Kit (#VPK-090), and the virus was used to infect isolated HSC cells using ViraDuctin™ Lentivirus Transduction Kit (#LTV-200).

**In vivo rat experiments**. The animal protocol conformed to US NIH guidelines (Guide for the Care and Use of Laboratory Animals, No. 85-23, revised 1996), and was reviewed and approved by the Institutional Animal Care and Use Committee from Wuhan University. The male Wistar rats were housed 4 or 5 per cage on a 12:12-h light-dark cycle and were given commercial rodent chow and water ad libitum on arrival.

Rat Protocol 1: Generation of diabetic rats. Chronic diabetic rats (2-month old) were induced by injection of 50 mg/kg streptozocin (STZ, 0.05 M sodium citrate, pH 5.5) after an 8-hour fasting period. The blood glucose was monitored one week after injection, the animals with blood glucose >300mg/dl consecutively for 3 days were considered positive, while control (CTL) rats received only vehicle injection (1, 2).

Rat Protocol 2: Rat models of cutaneous burn. The diabetic rats from Protocol 1 described above were subjected to a model of cutaneous burn injury after 2 weeks of STZ injection. The dorsum of each rat was shaved with electric clippers and depilated with Nair. The rats were anesthetized by inhalation of 5% isoflurane, and then the cutaneous burn injury was made on the dorsa of the rats by exposure to a hot copper pillar (2-cm diameter) at 75ºC for 15 seconds, and the subsequent wound healing process was monitored and evaluated (1, 2).

Rat Protocol 3: Treatments of rat models of cutaneous burns. The above rats from Protocol 2 received treatments of either vehicle (VEH), resveratrol (RSV), or pterostibene (PTE), which was first dissolved in 1% DMSO and then diluted 10 times in 0.9 % NaCl solution and was administered intraperitoneally every 3 days at a dose of 15 mg/(kg body weight) for 4 weeks starting from one week before the burn injury. The experimental rats were randomly separated into 4 groups as follows: Group 1: Control (CTL) rats that received vehicle (VEH) treatment (CTL/VEH); Group 2: STZ-induced diabetic (STZ) rats that received VEH treatment (STZ/VEH); Group 3: STZ-induced diabetic (STZ) rats that received RSV treatment (STZ/RSV); Group 4: STZ-induced diabetic (STZ) rats that received PTE treatment (STZ/PTE). During the treatment, the wound healing process was monitored and evaluated. After treatment, the rats were sacrificed and the hematopoietic stem cells (HSC) were isolated from the tibia and femur for either bone marrow transplantation (BMT) or biomedical analysis, including gene expression, ChIP analysis, SOD2 activity, oxidative stress, DNA damage and mitochondrial function. The peripheral blood mononuclear cells (PBMC) were also separated from blood using Ficoll-Paque Plus lymphocyte separation medium for further biomedical analysis.

Rat Protocol 4: Bone marrow transplantation (BMT) of HSC. The male rats (2-month old) were used as recipients for bone marrow transplantation (BMT). The HSC cells were isolated and characterized from rats in Protocol 1 (3), then the isolated HSC were purified by density centrifugation using Histopaque 1083^®^ (#-1083-1, Sigma) and then resuspended in 10ml of RPMI 1640 supplemented with 10% FBS and 2mM EDTA. The recipient male rats were lethally irradiated with 2 doses of 6 Gy 3 hours apart (4), and after 4 hours of irradiation, 2×10^6^ of isolated HSC cells were systemically transplanted by tail vein injection. All transplant-recipient rats were set aside for a minimum of 4 weeks to allow for complete reconstitution of the bone marrow (5) before they were then used for wound healing analysis. The experimental rats were randomly separated into 4 groups as follows: Rats with BMT of HSC from CTL/VEH (BMT-CTL/VEH); Rats with BMT of HSC from STZ/VEH (BMT-STZ/VEH); Rats with BMT of HSC from STZ/RSV (BMT-STZ/RSV); Rats with BMT of HSC from STZ/PTE (BMT-STZ/PTE). The above rats with BMT transplantation of HSC were subjected to a model of cutaneous burn injury for subsequent wound healing analysis.

Rat Protocol 5: Wound healing Measurement. Digital photographs of the wounds were taken every two days for 21 days. Wound area was quantified as a percentage area of the original wound size using Image J software. At indicated time points, wounds were excised and snap-frozen or, alternatively, processed for either H&E staining or immunohistochemistry (IHC). Images were taken using a Carl Zeiss MIRAX MIDI slide scanner, and the analyses were performed using a 3DHISTECH Pannoramic Viewer for the quantification of granulation tissue deposition (6). Vascular density was detected on frozen sections by IHC using CD31 mouse monoclonal antibody. For quantification of CD31 positivity, wounds were analyzed under 200ˣ magnification, and the number of positive 6 cells per high-power field (HPF) were counted. All counts and observations were performed by a blinded observer (7). Cytokine levels from rat serum were measured using ELISA kits from R&D Systems and the peritoneal macrophage was isolated for gene expression (1, 2, 7).

**Isolation and characterization of hematopoietic stem cells (HSC)**. The HSC preparation procedure is a minor modification from previously described method (3, 8). In brief, the whole bone marrow cells were collected from tibias in treated rats. Bone marrow cells were stained with antibodies for the identification of HSC (c-Kit^+^/Sca1^+^/Lineage^-^), and the following antibodies were used: c-Kit-PE (#sc-365504 PE from Santa Cruz Biotechnology), Sca-1-FITC, (react to rat, customized antibody from Dr Haimou Zhang, Hubei University), and anti-Lineage Antibody Cocktail comprises a mixture of PE-Cy5-conjugated antibodies, including anti-B220, anti-CD4, anti-CD8, anti-Gr-1, anti-Mac-1, and anti-TER119 (react to rat, customized antibodies from Dr Haimou Zhang, Hubei University). For HSC sorting, the debris, dead and clumped cells were firstly removed to obtain the single and viable cells, then the Sca-1 positive, c-Kit positive and Lineage negative cell population were isolated by HSC sorting, and the FACS analysis was performed on BD FACSMelody™ Cell Sorter.

**Isolation of rat PBMC cells**. The heparinized peripheral blood was collected from rats by puncturing the heart and was diluted 1:3 with Hank's balanced salts solution without Ca^2+^/Mg^2+^ (HBSS solution). The diluted blood was layered onto 15ml of Ficoll-Paque in 50ml sterile centrifuge tubes followed by centrifugation at 300×g at 20ºC for 40 min. The PBMC layers were then harvested and washed three times by HBSS solution. The pellets were then resuspended with lysing buffer containing 150mM NH4Cl, 1.0mM KHCO3, and 0.1mM Na2EDTA, pH 7.4 and incubated for 5 min at room temperature to remove contaminated red cells. The cell suspensions were then centrifuged and washed twice with HBSS solution, then the cell pellet was resuspended for further biomedical analysis.

**Isolation of rat peritoneal macrophages**. Macrophages were isolated from the peritoneal cavity of treated experimental rats. A 0.2 ml/ml solution of Concanavalin A was prepared in PBS and 1 ml was injected intraperitoneally into each rat. The rats were anesthetized using isoflurane 3 days after injection and a cardiac puncture was conducted to remove as much blood as possible. The abdominal skin was opened and 10 ml of warm PBS-PS (PBS plus 1% of penicillin and streptomycin) was injected intraperitoneally. After a gentle massage of the abdomen, a small incision was made in the abdominal wall to collect the fluid into a sterile 50ml conical tube. The abdominal cavity was then rinsed twice with warm PBS-PS and the collected fluid was centrifuged at 1,000 rpm for 5min. Sedimentary cells were resuspended with DMEM complete medium (containing 10% fetal bovine serum (FBS), 5mM glucose, 100U/ml penicillin, and 100 g/ml streptomycin) and adjusted to a required concentration, and then incubated in 37°C, 5% CO_2_ for 6 hr. Adherent cells were collected and cultured for 18 hr followed by subsequent analysis (9, 10).

**Wound macrophage isolation**. Wound tissue was harvested on day 15 after burn injury by 6-mm punch biopsy according to previous report with minor modifications. In brief, the wound tissues were digested at 37°C for 30 min with 50mg/ml Liberase (#5401020001 from Sigma) and 20 units/mL DNase I (#D4263 from Sigma). Samples were filtered over a 100-mm cell strainer to produce a single-cell suspension. Cells were then incubated with FITC (fluorescein isothiocyanate)-labeled anti-CD3, anti-CD19, and anti-Ly6G (BioLegend) followed by anti-FITC microbeads (Miltenyi Biotec). The flow-through was then incubated with anti-CD11b microbeads (Miltenyi Biotec) to isolate the non-neutrophil, non-lymphocyte, CD11b+ cells. Cells were then used to count the GFP positive cells under the fluorescence microscope (11).

**RT reaction and real-time quantitative PCR**. Total RNA from treated cells was extracted using the RNeasy Micro Kit (Qiagen), and the RNA was reverse transcribed using an Omniscript RT kit (Qiagen). All the primers were designed using Primer 3 Plus software with the Tm at 60°C, primer size of 21bp, and the product length in the range of 140-160bp (see Table S1). The primers were validated with an amplification efficiency in the range of 1.9-2.1 and the amplified products were confirmed with agarose gel. The real-time quantitative PCR was run on iCycler iQ (Bio-Rad) with the Quantitect SYBR green PCR kit (Qiagen). The PCR was performed by denaturing at 95°C for 8 min followed by 45 cycles of denaturation at 95°C, annealing at 60°C, and extension at 72°C for 10s, respectively. 1 µl of each cDNA was used to measure target genes. β-actin was used as the housekeeping gene for transcript normalization, and the mean values were used to calculate relative transcript levels with the ^ΔΔ^CT method per instructions from Qiagen. In brief, the amplified transcripts were quantified by the comparative threshold cycle method using β-actin as a normalizer. Fold changes in gene mRNA expression were calculated as 2^−ΔΔCT^ with CT = threshold cycle, ΔCT=CT (target gene)-CT(β-actin), and the ΔΔCT =ΔCT (experimental)-ΔCT (reference) (12, 13).

**Western Blotting.** The cells were lysed in an ice-cold lysis buffer (0.137M NaCl, 2mM EDTA, 10% glycerol, 1% NP-40, 20mM Tris base, pH 8.0) with protease inhibitor cocktail (Sigma). The proteins were separated in 10% SDS-PAGE and further transferred to the PVDF membrane. The membrane was incubated with appropriate antibodies, washed and incubated with HRP-labeled secondary antibodies, and then the blots were visualized using the ECL+plus Western Blotting Detection System (Amersham). The blots were quantitated by IMAGEQUANT, and the results were normalized by β-actin (13).

**Chromatin Immunoprecipitation (ChIP).** Cells were washed and crosslinked using 1% formaldehyde for 20 min and terminated by 0.1M glycine. Cell lysates were sonicated and centrifuged. 500µg of protein were pre-cleared by BSA/salmon sperm DNA with preimmune IgG and a slurry of Protein A Agarose beads. Immunoprecipitations were performed with the indicated antibodies, BSA/salmon sperm DNA and a 50% slurry of Protein A agarose beads. Input and immunoprecipitates were washed and eluted, then incubated with 0.2mg/ml Proteinase K for 2h at 42˚C, followed by 6h at 65˚C to reverse the formaldehyde crosslinking. DNA fragments were recovered by phenol/chloroform extraction and ethanol precipitation. A ~150bp fragment on the promoter was amplified by real-time PCR (qPCR) using the primers provided in Table S1 (12, 13).

**Measurement of ROS generation.** Treated cells were seeded in a 24-well plate and incubated with 10μM CM-H2DCFDA (Invitrogen) for 45 min at 37°C, and then the intracellular formation of reactive oxygen species (ROS) was measured at excitation/emission wavelengths of 485/530nm using a FLx800 microplate fluorescence reader (Bio-Tek). The data was normalized as arbitrary units (13, 14).

Measurement of DNA breaks. Comet assay was conducted using a CometAssay™ kit (Cat No. TA800) from R&D Systems Inc, and the 8-OHdG formation was measured using an OxiSelect™ Oxidative DNA Damage ELISA Kit (Cat No. STA320, from Cell Biolabs Inc.) according to manufacturers’ instructions. The formation of γH2AX was measured from nuclear extracts by western blotting using H2AX as input control.

**Evaluation of mitochondrial function.**

*Mitochondrial DNA copies.* The genomic DNA was extracted from cells using a QIAamp DNA Mini Kit (Qiagen) and the mitochondrial DNA was extracted using the REPLI-g Mitochondrial DNA Kit (Qiagen). The purified DNA was used for the analysis of genomic β-actin (marker of the nuclear gene) and ATP6 (ATP synthase F0 subunit 6, marker of the mitochondrial gene) respectively using the qPCR method mentioned above. The primers for genomic β-actin: forward 5’-acc aca gct gag agg gaa atc -3’ and reverse 5’- att gcc gat agt gat gac ctg-3’. The primers for ATP6: forward 5’- tag ggc ttc ttc ccc ata cat -3’ and reverse 5’- tta gtg aga tgg ggg ttc ctt-3’. The mitochondrial DNA copies were obtained from relative ATP6 copies that were normalized by β-actin copies using the ^ΔΔ^CT method.

*Intracellular ATP level.* The intracellular ATP level was determined using the luciferin/luciferase-induced bioluminescence system. An ATP standard curve was generated at concentrations of 10^-12^-10^-3^M. Intracellular ATP levels were calculated and expressed as nmol/mg protein(14).

*Measurement of apoptosis.* Apoptosis was evaluated by TUNEL assay using the In Situ Cell Death Detection Kit™ (Roche). Cells were fixed in 4% paraformaldehyde and labeled by TUNEL reagents. Stained cells were photographed by a fluorescence microscope and further quantified by FACS analysis. Caspase-3 activity was determined by the ApoAlert caspase assay kit (Clontech). Treated cells were harvested and 50 µg of proteins were incubated with the fluorogenic peptide substrate Ac-DEVD-7-amino-4-trifluoromethyl coumarin (AFC). The initial rate of free AFC release was measured using a FLx800 microplate reader (Bio-Tek) at excitation/emission wavelengths of 380/505nm, and enzyme activity was calculated as pmol/min/mg (14).

*Mitochondrial membrane potential (Δψm).* The Δψm was measured by TMRE (from Molecular Probes T-669) staining. 600μM T-669 stock solution was prepared using DMSO. Cells were grown on coverslips and immersed in 600nM TMRE for 20min at 37°C to load the cells with dye. The labeling medium was aspirated and cells were immersed in 150nM TMRE to maintain the equilibrium distribution of the fluorophore. Coverslips were mounted with live cells onto a confocal microscope to image the cells using 548nm excitation/573nm emission filters, and the intensity of TMRE fluorescence was measured using Image J software. Data from 10-20 cells were collected for each experimental condition and mean values of fluorescence intensity ± SEM were calculated.

**SOD2 activity assay**. SOD2 was obtained from the mitochondrial fraction that was isolated using a Pierce Mitochondria Isolation Kit (Pierce) according to manufacturers’ instructions. The successful isolation process was confirmed by the absence of TfR (transferrin receptor) protein in mitochondrial section and the absence of COXII (cytochrome c oxidase subunit II) protein in cytosolic fraction by western blots. SOD activity was measured as described previously (15). In brief, a stable O2^.-^ source was generated through the conversion action of XOD (xanthine oxidase) from xanthine and was mixed with chemiluminescent (CL) reagents to achieve a stable light emission. The SOD2 sample injection can scavenge O2.- and the subsequent decrease of chemiluminescent response is proportional to SOD2 activity. This system can have a detection limit of 0.001U/ml within the linear range of 0.03~2.00U/ml. The results were normalized by protein concentration and expressed as Units/mg proteins (U/mg) (16).

**Immunostaining**. The treated cells were transferred to cover slips and the cells were fixed in 4% paraformaldehyde for 20 min before being incubated with 0.3% Triton X-100 in PBS for 15 min. After blocking with 5% goat serum in PBS at room temperature for 30 min, cells were incubated with 8-oxo-dG anti-mouse antibody (# 4354-MC-050, from Novus Biologicals) for 12 h at 4°C and subsequently with secondary antibody Alexa Fluor 488. The cover slips were then mounted by antifade Mountant with DAPI (staining nuclei, in blue). The photographs were taken using a [Confocal Laser Microscope](https://www.sogou.com/link?url=DSOYnZeCC_qw-OVKG_MsR3KENashJ6PPMhOejy_Q5JJflCntg_rzjU2lo9-QKkufX5Qp7YP6841C08P_Gzn4lQD4cR4JDdkk5sef3Ee0PfoOX3hBKf-DUA..) (Leica, 20x lens) and quantitated by Image J. software (17).

# **ELISA.** Rat interleukins from either supernatant or serum, including IL1β, IL6 and MCP1, were measured by Rat IL-1β/IL-1F2 Quantikine ELISA Kit (#RLB00), Rat IL-6 Quantikine ELISA Kit (#RRA00), and Rat JE/MCP-1/CCL2 DuoSet ELISA Kit (#DY3144-05), respectively, according to the manufacturer’s instructions from R&D Systems (18).

**Immunohistochemistry.** The tissues were dissected and snap-frozen in the OCT compound. The 10μm sections were cut by clean microtome and mounted on PEN-membrane slides (2.0μm, Leica), and stored at -20ºC before use. The slides were first fixed by 3.7% formaldehyde at 37ºC for 15 min, permeabilized by 1% BSA+0.2% Triton X-100 in PBS for 1 hour, and then blotted with 40μg/ml (dilute 1:20) of either MCP1, or CD31 mouse monoclonal antibody for 2 hours. They were then washed three times and the Texas-red (for CD31) or DAB (for MCP1) labeled anti-mouse secondary antibody (1:200) was added for blotting for another 1 hour. After thorough washing, the slides were visualized and photographed. The relative densities of each group were quantitated for protein expression using Image J. software (1, 2, 19).

**In vivo superoxide release**. Superoxide anion (O_2_^.-^) release from the tissue was determined by a luminol-EDTA-Fe enhanced chemiluminescence (CL) system supplemented with DMSO-TBAC (Dimethyl sulfoxide-tetrabutyl-ammonium chloride) solution for extraction of released O_2_^.-^ from tissues as described previously (14). The superoxide levels were calculated from the standard curve generated by the xanthine/xanthine oxidase reaction (1, 2, 13).

**Statistical analysis**. The data was given as mean ± SEM; all of the experiments were performed at least in quadruplicate unless otherwise indicated. The one-way ANOVA followed by the Bonferroni post hoc test was used to determine statistical significance of different groups. SPSS 22 software was used for statistical analysis and a *P* value < 0.05 was considered significant (1, 2).

REFERENCES

1. Li M, Yu H, Pan H, Zhou X, Ruan Q, Kong D, et al. Nrf2 Suppression Delays Diabetic Wound Healing Through Sustained Oxidative Stress and Inflammation. *Front Pharmacol.* 2019;10:1099.

2. Zhou X, Li M, Xiao M, Ruan Q, Chu Z, Ye Z, et al. ERβ Accelerates Diabetic Wound Healing by Ameliorating Hyperglycemia-Induced Persistent Oxidative Stress. *Front Endocrinol (Lausanne).* 2019;10:499.

3. Rossi L, Challen GA, Sirin O, Lin KK, and Goodell MA. Hematopoietic stem cell characterization and isolation. *Methods Mol Biol.* 2011;750:47-59.

4. Xie W, Ren M, Li L, Zhu Y, Chu Z, Zhu Z, et al. Perinatal testosterone exposure potentiates vascular dysfunction by ERbeta suppression in endothelial progenitor cells. *PLoS One.* 2017;12(8):e0182945.

5. Ii M, Nishimura H, Iwakura A, Wecker A, Eaton E, Asahara T, et al. Endothelial progenitor cells are rapidly recruited to myocardium and mediate protective effect of ischemic preconditioning via "imported" nitric oxide synthase activity. *Circulation.* 2005;111(9):1114-20.

6. Zhang H, Li L, Chen Q, Li M, Feng J, Sun Y, et al. PGC1beta regulates multiple myeloma tumor growth through LDHA-mediated glycolytic metabolism. *Mol Oncol.* 2018;12(9):1579-95.

7. Thangarajah H, Yao D, Chang EI, Shi Y, Jazayeri L, Vial IN, et al. The molecular basis for impaired hypoxia-induced VEGF expression in diabetic tissues. *Proc Natl Acad Sci U S A.* 2009;106(32):13505-10.

8. Yan J, Tie G, Wang S, Tutto A, DeMarco N, Khair L, et al. Diabetes impairs wound healing by Dnmt1-dependent dysregulation of hematopoietic stem cells differentiation towards macrophages. *Nat Commun.* 2018;9(1):33.

9. Yu T, Gao M, Yang P, Liu D, Wang D, Song F, et al. Insulin promotes macrophage phenotype transition through PI3K/Akt and PPAR-gamma signaling during diabetic wound healing. *J Cell Physiol.* 2019;234(4):4217-31.

10. Ray A, and Dittel BN. Isolation of mouse peritoneal cavity cells. *J Vis Exp.* 2010(35).

11. Kimball AS, Joshi A, Carson WFt, Boniakowski AE, Schaller M, Allen R, et al. The Histone Methyltransferase MLL1 Directs Macrophage-Mediated Inflammation in Wound Healing and Is Altered in a Murine Model of Obesity and Type 2 Diabetes. *Diabetes.* 2017;66(9):2459-71.

12. Zou Y, Lu Q, Zheng D, Chu Z, Liu Z, Chen H, et al. Prenatal levonorgestrel exposure induces autism-like behavior in offspring through ERbeta suppression in the amygdala. *Mol Autism.* 2017;8:46.

13. Zhang H, Li L, Li M, Huang X, Xie W, Xiang W, et al. Combination of betulinic acid and chidamide inhibits acute myeloid leukemia by suppression of the HIF1alpha pathway and generation of reactive oxygen species. *Oncotarget.* 2017;8(55):94743-58.

14. Yao D, Shi W, Gou Y, Zhou X, Yee Aw T, Zhou Y, et al. Fatty acid-mediated intracellular iron translocation: a synergistic mechanism of oxidative injury. *Free Radic Biol Med.* 2005;39(10):1385-98.

15. Yao D, Vlessidis AG, Gou Y, Zhou X, Zhou Y, and Evmiridis NP. Chemiluminescence detection of superoxide anion release and superoxide dismutase activity: modulation effect of Pulsatilla chinensis. *Anal Bioanal Chem.* 2004;379(1):171-7.

16. Kong D, Zhan Y, Liu Z, Ding T, Li M, Yu H, et al. SIRT1-mediated ERbeta suppression in the endothelium contributes to vascular aging. *Aging Cell.* 2016.

17. Wang X, Lu J, Xie W, Lu X, Liang Y, Li M, et al. Maternal diabetes induces autism-like behavior by hyperglycemia-mediated persistent oxidative stress and suppression of superoxide dismutase 2. *Proc Natl Acad Sci U S A.* 2019;116(47):23743-52.

18. Kobayashi EH, Suzuki T, Funayama R, Nagashima T, Hayashi M, Sekine H, et al. Nrf2 suppresses macrophage inflammatory response by blocking proinflammatory cytokine transcription. *Nat Commun.* 2016;7:11624.

19. Li H, Liu Z, Gou Y, Yu H, Siminelakis S, Wang S, et al. Estradiol mediates vasculoprotection via ERRalpha-dependent regulation of lipid and ROS metabolism in the endothelium. *J Mol Cell Cardiol.* 2015;87:92-101.

**Table S1. Sequences of primers for the real time quantitative PCR (qPCR)**

| Gene | Species | Analysis | Forward primer (5'→3') | Reverse primer (5'→3') |
| --- | --- | --- | --- | --- |
| ERβ | Rat | ChIP | gggtgtccctagtggatgact | aaaagagtgtgggagggtagc |
| β-actin | Rat | mRNA | ttccttcctgggtatggaatc | cttctgcatcctgtcagcaat |
| ERβ | Rat | mRNA | tcagcatgaagtgcaaaaatg | ggttctgggagctctctttgt |
| NRF1 | Rat | mRNA | gtttcatggacccaagcatta | gtgatggtacgagatgggcta |
| SOD2 | Rat | mRNA | caactcaggttgctcttcagc | ctcaaaagacccaaagtcacg |
| IL1β | Rat | mRNA | gagagtgtggatcccaaacaa | ggaagacaggtctgtgctctg |
| IL6 | Rat | mRNA | agccagagtcattcagagcaa | gtcttggtccttagccactcc |
| MCP1 | Rat | mRNA | tcacctgctgctactcattca | attccttattggggtcagcac |

FIGURE S1

**Figure S1. Representative pictures of full blots for Western Blotting.** (a) Representative full blots for Figure 1f. (b) Representative full blots for Figure 1j. (c). Representative full blots for Figure 2c. (d). Representative full blots for Figure 3e. (e). Representative full blots for Figure 5c.

FIGURE S2

**Figure S2.** **Bone marrow transplantation (BMT) of GFP lentivirus infected-HSC cells can differentiate into PBMC and macrophage cells.** The HSC cells from donor rats were collected and infected by GFP lentivirus for bone marrow transplantation (BMT) to recipient rats, and the GFP positive cell rate was counted. After 4 weeks of BMT, the PBMC and wound macrophage cells were isolated and the GFP positive cells were counted. (a) Representative GFP pictures for 3 different treatments, including HSC before BMT, PBMC after BMT and wound macrophages after BMT. (b) Quantitation of GFP positive cells for (a), n=5. *, *P*<0.05, vs HSC before BMT group. Data were expressed as mean ± SEM.

FIGURE S3

**Figure S3.** **Bone marrow transplantation (BMT) of treated HSC has no effect on the gene expression of ERβ and its target genes from local wound tissues.** The experimental rats were randomly separated into 4 groups as follows: Rats with BMT of HSC from CTL/VEH (BMT-CTL/VEH); Rats with BMT of HSC from STZ/VEH (BMT-STZ/VEH); Rats with BMT of HSC from STZ/RSV (BMT-STZ/RSV); Rats with BMT of HSC from STZ/PTE (BMT-STZ/PTE). The above BMT rats were subjected to a model of cutaneous burn injury, and the mRNA expression of ERβ and its target genes from local wound tissues was measured, n=4. Data were expressed as mean ± SEM.
